# Sluggish cognitive tempo and its neurocognitive, social and emotive correlates: a systematic review of the current literature

**DOI:** 10.1186/2049-9256-2-5

**Published:** 2014-08-05

**Authors:** Anna Katharina Mueller, Lara Tucha, Janneke Koerts, Yvonne Groen, Klaus W Lange, Oliver Tucha

**Affiliations:** Department of Clinical and Developmental Neuropsychology, University of Groningen, Groningen, the Netherlands; Department of Experimental Psychology, University of Regensburg, Regensburg, Germany

**Keywords:** Sluggish cognitive tempo, ADHD, Genetics, Cognition, Social functioning, ADHD subtypes

## Abstract

**Objectives:**

Since the elimination of items associated with Sluggish Cognitive Tempo (SCT) during the transition from DSM-III to DSM-IV from the diagnostic criteria of Attention-deficit Hyperactivity Disorder (ADHD), interest in SCT and its associated cognitive as well as emotional and social consequences is on the increase. The current review discusses recent findings on SCT in clinical as well as community based ADHD populations. The focus is further on clinical correlates of SCT in populations different from ADHD, SCT’s genetic background, SCT’s association with internalizing and other behavioral comorbidities, as well as SCT’s association with social functioning and its treatment efficacy.

**Method:**

A systematic review of empirical studies on SCT in ADHD and other pathologies in PsycInfo, SocIndex, Web of Science and PubMed using the key terms “Sluggish Cognitive Tempo”, “Cognitive Tempo”, “Sluggish Tempo” was performed. Thirty-two out of 63 studies met inclusion criteria and are discussed in the current review.

**Results/Conclusion:**

From the current literature, it can be concluded that SCT is a psychometrically valid construct with additive value in the clinical field of ADHD, oppositional defiant disorder (ODD), internalizing disorders and neuro-rehabilitation. The taxonomy of SCT has been shown to be far from consistent across studies; however, the impact of SCT on individuals’ functioning (e.g., academic achievement, social interactions) seems remarkable. SCT has been shown to share some of the genes with ADHD, however, related most strongly to non-shared environmental factors. Future research should focus on the identification of adequate SCT measurement to promote symptom tailored treatment and increase studies on SCT in populations different from ADHD.

**Electronic supplementary material:**

The online version of this article (doi:10.1186/2049-9256-2-5) contains supplementary material, which is available to authorized users.

## Review

### Introduction

The current literature review gives an overview about the research performed on the concept of Sluggish Cognitive Tempo (SCT). SCT is a cognitive-emotional style that is commonly described by five typical characteristics, which are “daydreaming”, “being confused”, “staring blankly”, “being sluggish” and “being unmotivated” [1, 2]. SCT was originally introduced in the literature on ADHD but is nowadays recognized in disorders different from ADHD as well [3–5]. The Diagnostic and Statistical Manual of Mental Disorders (DSM-IV and DSM-5) distinguishes three different subtypes of ADHD, namely ADHD combined type (ADHD/C), ADHD predominantly inattentive (ADHD/I) or ADHD predominantly hyperactive/impulsive (ADHD/HI) [6]. The transition from the 3rd to the 4th edition of the Diagnostic and Statistical Manual of Mental Disorders [7], however, led to the removal of items representing sluggishness, easy confusion, and daydreaming from the inattention dimension of ADHD [8] due to poor predictive validity [9]. Regardless of the increasing interest in symptoms of sluggishness in ADHD during the last two decades [10–13] the current DSM-5 has not reintroduced the items representing a sluggish cognitive-emotive style. Based on recent psychometric findings, however, it is argued that the elimination of SCT symptoms during the transition from the DSM-III to the DSM-IV artificially increased ADHD’s homogeneity [14, 15]. As a consequence, it is hypothesized that some of the individuals who actually would have met diagnostic criteria of one of the ADHD subtypes are missed due to the elimination of SCT items.

Especially, ADHD/I has been frequently linked to symptoms such as, daydreaming, staring, mental fogginess, confusion, hypoactivity, sluggish or slow movement, lethargy, apathy and sleepiness [6, 15–19]. It is striking that the mentioned symptoms are very similar to items currently used in the measurement of SCT. In line with this, 30 to 50% of the children diagnosed with ADHD/I have been shown to present with increased levels of symptoms that emerged under the label SCT [20]. Based on the observation that approximately twice as many school-aged children are nowadays diagnosed with ADHD/I in contrast to ADHD/HI [21, 22] and ADHD/I’s strong association with SCT, there is a need for a thorough definition of SCT’s cascading effects on individuals’ functioning. This being said, the current literature on SCT is rather inconsistent in terms of the definition and measurement of SCT. No consensus has been met, yet, with regard to symptomatology or standardization in the assessment of SCT. Neurocognitive impairments that are seen in children with ADHD/I with comorbid SCT but not in children with pure ADHD/I further show that even though SCT is very similar to ADHD/I, SCT has its own neurocognitive characteristics [6, 17, 23, 24]. A thorough look at the diagnostic validity of SCT and its impact on a variety of individuals’ functional domains seems therefore to be warranted. Furthermore, given the heterogeneity in the measurement of SCT and its inconsistency in the definition of the concept SCT, treatment approaches of SCT are, so far, rather sparse. Yet, given the outlined neurocognitive characteristics that are typical for SCT but not ADHD [6, 17, 23, 24], treatment that is independent of a possible comorbidity of ADHD seems to be important. The presence of SCT symptoms above and beyond ADHD symptoms might be one of the mediating factors in treatment efficacy in psychiatrically referred individuals.

During the process of the review another review on the same topic was published [25]. In contrast to Becker [25] the current review discusses findings on SCT in adults, genetic studies, gender differences, SCT in disorders different than ADHD [3–5], specific treatment of SCT and stresses the current lack of standardization in the assessment of SCT. Each section of this review will be dedicated to one of the functional domains that have been shown to be affected in individuals with SCT. It was our aim to dissect the unique contribution of SCT to impairments, whenever the reviewed studies’ designs allowed for such a conclusion.

## Method

A systematic review of the English published literature of several databases (PsycInfo, SocIndex, Web of Science, PubMed) on the key terms “Sluggish Cognitive Tempo”, “Cognitive Tempo”, “Sluggish Tempo” revealed a total of 63 articles of which 32 (see Additional file 1: Table S1. Studies and measures employed) were closely related to SCT and will be reviewed in here. Inclusion criteria were: SCT was measured by questionnaire or observation and its relation to neuropsychological, emotional or social functioning was tested. The remaining 31 studies were not included in this review since SCT was not systematically assessed or the focus was on age-related cognitive slowing or learning (e.g., reading) ability and its relation to cognitive tempo. The majority (92%) of the studies focused on SCT comorbid to ADHD symptoms as a personality trait or clinical disorder.

## Literature review

### Lack of standards in the measurement of SCT

Current studies on SCT predominantly focused on SCT in children and/or adolescents with either traits [11, 26] or clinical diagnoses of ADHD [10, 27]. Only one study looked into SCT in adults with ADHD, examining hereby the association between self-rated SCT and executive functioning [16]. Initially, SCT was represented by four items (“difficulty following instructions”, “sluggishness”, “drowsiness”, “absent-minded, forgetful” [28]) that were then either reduced to two (“daydreams”, “is low of energy” [12, 17, 29–31]) or expanded up to 17 items in more recent studies [19, 32, 33].

Penny and colleagues [2] addressed the lack of agreement in standardised measures in SCT and came up with a unique SCT questionnaire based on items that have been shown to load highly on SCT in previous research [14, 19]. An extensive review of the literature on available items measuring SCT with subsequent reliability and factor structure analysis decreased the initial pool of 26 items to a 14-item SCT scale (see Additional file 1: Table S1 for individual items; [2]). In contrast to Penny’s 14 item scale, Skirbekk and colleagues [33] compared the utility of Pfiffner and colleagues’ [32] 17-item scale (SCT-17 see Additional file 1: Table S1 for individual items) to a 5-item scale (SCT-5 see Additional file 1: Table S1 for individual items) by Hartman and colleagues [14] and showed that both scales capture the concept of SCT [23, 27, 34] but add to Penny and colleagues’ scale the dimensions of confusion [14] or forgetfulness (SCT-15 [32]). According to Penny and colleagues [2], the items measuring the concept of confusion (i.e., forgetfulness, disorganization and difficulty following instructions) were explicitly removed as they are part of the ADHD DSM-IV criteria and nowadays DSM-5 criteria of inattention. Moreover, Hartman and colleagues [14] showed that their five SCT items (SCT-5) loaded strongly on the same factor that was identified to represent cognitive and physiological sluggishness by the more extensive Child Behavior Checklist (CBCL [35]), contributing further evidence of adequate convergent validity of their 5-item SCT scale. The presented findings indicate that it appears not to be the number of items that matter in measuring SCT but the items’ representativeness, with the short 5-SCT scale of Hartman and colleagues [14] being a promising tool in diagnosing SCT in the pediatric setting. Whether the 5-SCT scale by Hartman and colleagues [14] would outweigh the utility of Penny and colleagues’ [2] 14-SCT scale is yet to be investigated.

To conclude, even though various scales have been shown to effectively measure SCT [16, 31, 36], a standard of measurement across studies is not yet achieved. Moreover, some evidence points into the direction that even though SCT items might identify individuals with SCT [2, 11, 14], their contribution in distinguishing subtypes of ADHD should be questioned [13, 30]. Independent of the number of items that were used to represent SCT, “daydreaming”, “sluggish/drowsy” and “underactive/apathetic” were items that consistently contributed to the identification of SCT in children [2, 11, 14, 19] and adults [16]. More in detail, the item “daydreaming” was represented in all studies reviewed [23, 27, 29, 34] followed by “sluggish/drowsy” [16, 30, 31, 36] and “underactive/apathetic” [11, 16, 23, 37]. Future research could use these three items as a baseline measurement for SCT without missing out to assess their individual link to behavioral and neurocognitive correlates.

A consequence of the lack of standardization in SCT measurement reinforced the discussion about whether SCT is a disorder itself above and beyond ADHD [6, 11, 16, 19], or whether SCT is comorbid to, or a subtype of ADHD [13, 17, 36, 38]. The following section is dedicated to this issue and will give more insight into the comorbidities of SCT.

### SCT and its link to ADHD subtypes

To start with, not only SCT is questioned for its diagnostic value in and above ADHD but so is ADHD itself. One of the current debates concerning ADHD focuses on the question whether ADHD should be handled as a continuum or as a clear-cut category of behavioral, cognitive and emotional deficits [1, 30]. Supporters of the former thesis welcomed the increase in studies focusing on SCT in ADHD and tested the contribution of SCT in diagnosing ADHD [1, 30]. Evidence was found that SCT not only enhances the reliability of diagnosing ADHD [19, 30, 36] but also contributes to the identification of a new subtype of ADHD [2] or a disorder itself [6, 11, 16, 19]. It is currently hypothesized that the SCT construct captures attentional deficits that are not represented by the nine symptoms measuring inattention according to the DSM-IV (as well as DSM-5) guidelines for ADHD/I [19, 33]. The endorsement of *sleepy/sluggish* and *slow/daydreamy* symptoms were shown to be more likely to be associated with ADHD/I than with ADHD/C or ADHD/HI [1, 10, 13, 17, 23, 29, 38], supporting the idea that the inclusion of the SCT items in diagnosing ADHD might enhance the reliability of ADHD/I diagnoses [19, 36]. In line with this, SCT subscales have been shown to present with both, good discriminant validity with symptoms of hyperactivity and strong convergent validity with symptoms of inattention [2]. McBurnett and colleagues [19] showed that when SCT symptoms were tested in ADHD/I [1, 10, 36], the SCT items did not show the extremely poor loading that ultimately led to the exclusion of SCT items from the DSM-IV [8, 9]. It can be concluded, that the inclusion of SCT items in the identification of individuals with ADHD/I seems therefore valuable. At the same time, it can be reasoned that when SCT contributes to a more defined subtyping of ADHD/I it also (indirectly) enhances the number of ADHD diagnoses. This is in line with the former proposed argument that excluding SCT from ADHD criteria might lead to missing out on individuals that would otherwise be diagnosed with ADHD/I [19, 36].

In contrast to accumulating evidence that SCT item inclusion enhances the number of ADHD/I diagnoses [13, 17, 38], no difference in SCT were found in children with ADHD/I and ADHD/C [13, 27, 33] or in class room observations of children with behaviour of ADHD [30, 34]. Expanding the association of SCT with ADHD or ADHD/I to ADHD common comorbidities, Skirbekk and colleagues [33] found that children with anxiety comorbid to ADHD exhibited the highest levels of SCT, followed by children with exclusively ADHD and finally children with clinical levels of anxiety compared to children with neither anxiety nor ADHD [33]. Unfortunately, children were not further subtyped into ADHD/I, ADHD/C or ADHD/HI, complicating thereby the comparison of findings across studies. Nevertheless, the presence of the highest SCT symptoms in children with anxiety and ADHD supports the idea that not merely ADHD is affected by SCT but SCT might be present in ADHD comorbidities in general and emotional disorders in particular. Further evidence that SCT can be found in disorders different from ADHD is given in the following section.

### SCT is not ADHD but a disorder distinct from ADHD

SCT has been shown to not only be present in children with ADHD [2, 33] but also in children who do not meet ADHD criteria [16]. Findings that SCT and ADHD/I relate differently to symptoms of inattention [6, 11, 19] underline the idea that SCT is a disorder itself and not merely comorbid to ADHD/I. Moreover, SCT emerged as a separate psychometric valid construct during data analysis in children that did not reach ADHD thresholds of DSM-IV criteria but presented with clinical symptoms of behavioural/emotional and/or learning difficulties [13]. The idea that SCT might relate to attentional impairments in clinical groups in general led Reeves and colleagues [4] to assess the presence and relationship of SCT to later cognitive outcomes of pediatric survivors of acute lymphoblastic leukemia. Lymphoblastic leukemia survivors were successfully identified from their healthy siblings by SCT. Moreover, SCT related to the survivors’ intellectual and achievement deficits [4]. Similarly, SCT was found in children diagnosed with Fetal Alcohol Syndrome (FAS) with and without comorbid ADHD and in children with clinically behavioral deviance [3]. SCT was found to be statistically linked but distinct from ADHD in a large sample of clinically distressed children [5]. The items “being confused”, “daydreaming”, and “stares blankly into space” related to internalizing and social deficits, as well as behavioral problems independently of ADHD or other psychopathologies in psychiatrically hospitalized children [5]. It can be concluded that SCT is not only a valuable factor in the clinical assessment of children or adolescents with ADHD but might be a valuable factor to look at in pathologies different from ADHD [3, 4, 13].

### SCT etiology: environment vs. genetics

Based on the observation that SCT was typically associated with ADHD, and ADHD has been shown to be highly heritably (additive and dominant genetic effects of around 75% for ADHD; [39]), researchers became interested in SCT’s genetic background. A recent twin study showed that the association between SCT and ADHD/I was almost twice as strong as the association between SCT and ADHD/HI [26]. While genetic factors were shown to be of particular significance in hyperactive-impulsive behaviour in general, the non-shared environmental factors were the major factor likely to explain individual differences in SCT [26]. Accordingly, the association of SCT with ADHD/I was found to be partly due to genetic (r = 0.29) and partly due to non-shared environmental factors (r = 0.21), whereas the association of SCT with ADHD/HI was almost purely attributable to genetic factors [26]. These findings indicate that SCT, even though it is genetically related to ADHD, is the least heritable subtype among ADHD [26]. SCT’s special association with environmental factors, led the authors to suggest that SCT might develop due to the environment created by facing ADHD symptoms [26]. Moruzzi and colleagues’ pioneering work should be carried on in a population in which SCT symptoms are the main reason for being referred to a clinician. If similar neurocognitive and or behavioural SCT symptoms exist between individuals who are seen for their SCT symptoms only and those with other comorbid conditions (such as ADHD or other pathologies), symptoms should be further tested for their genetic vs. (psychopathological-) environmental background.

### SCT’s cognitive and neuropsychological correlates

#### Processing speed

SCT’s association to various cognitive and neuropsychological correlates has been one of the major interests in the last decade of research in the field of ADHD. Whereas earlier studies suggested that children with SCT present with slow motor and processing speed [6, 15, 17, 28, 38] more recent studies could not replicate a link between SCT and processing speed [11]. The studies which reported such a link [15, 17, 28, 38] are based on the assessment of individuals with ADHD/I that are characterized by high levels of SCT, whereas Bauermeister and colleagues [11] focused on individuals with pure SCT and its impact on information processing. Furthermore, the majority of studies reporting an association between SCT and slowed information processing [15, 17, 28] did not make use of neuropsychological/behavioural assessments of cognitive tempo, but refer to teacher and parent observations only (e.g., the child seems to be “lost in a fog”, “daydreaming or getting lost in thought,” and “apathetic or unmotivated”). It has to be questioned whether behavior observations reliably reflect slow processing speed or whether reductions of processing speed are better depicted by actual assessments using psychometrically valid information processing tasks, such as reaction time measures, visual search- and pattern recognition tasks, or perceptual timing tasks. Experimental data indeed confirmed that children with ADHD/I and SCT presented with a slower task accomplishment during the Tower of London Task (ToL) and higher mean reaction times in the Continuous Performance Test [31, 40]. The authors concluded that children with ADHD/I and SCT do not present with inaccurate performance but do perform neurocognitive tasks in a conspicuous slow tempo [31]. It has therefore been hypothesized that SCT does not affect the underlying cognitive function per se (e.g., EF, inhibitory control) but compromises the overall task performance by slowing down task related processes [31].

#### Attention

Solanto and colleagues [31], however, did not control in their study for the children’s attentional functioning. Especially deficits in sustained attention have been found to be related to SCT [24, 27, 33]. The reason why some studies failed to confirm attentional deficits in children with SCT [17, 41] might again be that subjective teacher ratings based on class-room observations are not sufficiently sensitive and valid and by this miss relevant aspects of the children’s attentional problems. While studies applying neuropsychological tasks, such as classic vigilance, divided attention and selective attention tasks, revealed no differences in attention between ADHD subtypes [41–43], a unique association was found between SCT and early selection deficits in a perceptual load paradigm [41]. For an overview of the exact neuropsychological tests applied, please view Additional file 1: Table S1. Children with ADHD and comorbid SCT showed more interference on early selection tasks than children with ADHD but without SCT [41]. Deficits in sustained attention of individuals with SCT were further explained by an increased variability in spatial memory performance [33]. It seems likely that other functions, such as EF or inhibitory control as reported above (see discussion of [31]), might also be deviant due to sustained attention deficits rather than processing speed.

#### Executive functioning

Barkley [16] recently showed that SCT symptoms explained unique variance of self-rated executive functioning (EF) independent of the impairments associated with ADHD/I or ADHD/HI. These results, however, could not be replicated in a younger sample of children scoring high on ADHD and SCT [11]. Furthermore, behavioural ratings of EF in adolescents were also found to be unrelated to SCT but associated with ADHD/HI symptomatology [5]. These inconsistencies in findings might result from different assessment strategies applied (self-rated vs. observer-rated) and different age groups assessed (children vs. adolescents vs. adults) in the mentioned studies [5, 11, 16]. With regard to the differences in age between the samples it has to be considered that EF is known for its developmental trajectory [45]. More in detail, EF was shown to be relatively mature at the age of 12 but knows a transitional period of development at the beginning of adolescence [45]. It therefore can be speculated that the adult sample of Barkley [16] was much more aware of their EF deficits and, hence, more likely to report them than the observer-rated younger participants of Bauermeister and colleagues [11] and Becker and colleagues [5]. Furthermore, previous research has shown that EF self-ratings are more sensitive to particular EF related symptoms than neuropsychological tests [46]. However, as Barkley [16] did not control for the impact of comorbid ADHD subtypes it remains open whether the association between SCT and EF was unaffected by the underlying ADHD symptomatology.

#### Summary

Studies that controlled for ADHD/I symptomatology in order to show the unique impact of SCT on attentional functioning support the idea that SCT is associated with more severe attention deficits than ADHD/I [27]. Moreover, SCT related to sustained attention deficits that were not seen in ADHD/I [24]. The established link between SCT and attention deficits [24, 41] might lead to the suggestion that individuals with SCT are in general more prone than individuals with ADHD to perform worse on neurocognitive tasks and experience more deficits in everyday functioning. This knowledge appears relevant for the treatment of SCT because attentional functioning has been shown to be one of the core factors predicting rehabilitation outcome [47, 48], including treatment efficacy and socio-cognitive functioning in general [47, 48]. Research confirming a link between SCT and poor cognitive outcome [4, 13] further underlines the need for research on SCT in neuro-rehabilitation populations. To support this, SCT has been proposed to be the behavioral manifestation of slow processing speed [4], with slow processing speed being one of the rather common cognitive late effects after acquired or developmental neurocognitive pathologies in general [8]. Research on SCT in neuro-rehabilitation populations seems therefore warranted.

### SCT and internalizing symptoms: SCT’s relation to depression and anxiety

Lahey [28] was one of the first who reported differences in comorbidities according to ADHD subtypes. Whereas ADHD/C is more likely to be associated with externalizing behavior, ADHD/I appears more often to be linked to internalizing symptoms, with both types of ADHD also being related to conduct disorder (CD) [28]. After removing children with CD from their analysis, cognitive tempo was the most distinguishing factor between ADHD/C and ADHD/I [28]. Lahey [28] therefore concluded that except for the cognitive tempo factor, other symptom differences in ADHD might be due to other comorbidities than SCT, such as externalizing and internalizing. Given that SCT is foremost believed to be associated with ADHD/I, SCT’s proneness to internalizing behavior seems to be comprehensible.

Consequently, recent research confirms the link between SCT and internalizing symptoms such as anxiety and depression [2, 8, 11, 13, 17, 37], with some authors claiming that SCT is stronger related to depression than to anxiety [5, 36] even though correlations of SCT with depression were rather modest [36]. Comorbidity of internalizing disorders was based on validated rating scales such as the DISC-IV [5, 37], the Emory Combined Rating Scale (ECRS) [27] or the Impairment Rating Scale (IRS) [36]. Moreover, clinically referred children with severe SCT presented with increased symptoms of depression, a greater risk for generalized anxiety, social phobia and obsessions than children with low SCT [27]. The authors concluded that SCT is more likely to go along with internalizing disorders, such as depressive disorders and generalized anxiety disorders, than ADHD [27]. However, SCT has been shown not to be merely a measure of depression but to be statistically distinct from depression [5, 36]. Furthermore, internalizing problems correlated with SCT independently of ADHD inattention problems [11], leading to the assumption that SCT knows its own internalizing dimension that is not linked to ADHD symptomatology [2, 11].

With regard to anxiety it has been shown that the association between SCT and symptoms of anxiety is mediated by symptoms of inattention [33]. The authors hypothesized that anxiety disorder comorbid to ADHD decreases individuals’ attentional capacities even further [33]. The decrease in attentional capacities by anxiety is further believed to make individuals more vulnerable to leave the impression of being sluggish and slow [33]. However, it seems likewise probable that ADHD with comorbid SCT makes individuals more prone to develop a depressive or anxiety disorder [27, 33]. For example, a similar association between SCT and internalizing behavior was observed in clinical populations suffering from ADHD and SCT [33] as well as in populations with SCT and a clinical condition different from ADHD (FAS [3]). Furthermore, anxiety and depressive symptoms of clinically-distressed children correlated with levels of SCT, even beyond the ADHD/ODD symptomatology [5]. Notably, SCT levels were found to be stronger related to depression than to anxiety symptoms when parent-ratings were controlled for the parents’ own anxiety and depressive symptoms [5]. Becker and colleagues’ findings indicate that the raters own mental constitution should be taken into consideration when interpreting results from ratings that are not directly derived from the target population (e.g., no self-rating measures).

In contrast, the findings of studies focusing on SCT’s impact on externalizing, disruptive behavior, which is often seen in ODD, are rather promising [24]. For example, Wåhlsted and colleagues [24] showed that behavioral symptoms of SCT were not associated with internalizing problems but showed an interaction with inattention and ODD in a community sample of children. In more detail, more severe SCT decreased the likelihood of ODD symptoms in children with very distinct symptoms of ADHD symptoms [24, 27]. Furthermore, high levels of SCT were shown to be related with low levels of disruptive behavior [2], making SCT a protective factor against ODD symptomatology in children with ADHD [17, 20, 23].

#### Summary

It can be concluded that even though SCT seems to be related with internalizing, emotive disorders, its unique interaction with other comorbidities should be disentangled first. Moreover, whereas SCT can be interpreted as a risk factor per se, making individuals more prone to depression, SCT has also been shown to lower individuals’ risks of severe disruptive behavior.

### SCT and other characteristics: SCT’s link to academic functioning, gender, age-of-onset

SCT has been demonstrated to be further associated with academic outcomes in general [10], math [11], linguistic processing deficits [49] and initiative taking as well as motivation in particular [10, 36]. It has been shown that especially low levels of initiation and persistence in clinically referred children with ADHD and SCT contribute to the impairments seen in academic achievements [36]. Furthermore, math scores did not only negatively relate to ADHD symptoms of inattention but also to SCT [11]. It was shown that SCT beyond symptoms of inattention and hyperactive-impulsive symptoms relate to academic achievements [11, 50]. However, when relying on parent ratings of SCT, a link between SCT and academic achievements as was seen in teacher ratings [11, 50] could not be confirmed [51]. The observation that teacher and parent ratings of SCT yield different results with respect to SCT’s impact on academic functioning calls for thorough investigation of to what extent findings on SCT and its correlates are affected by within- (SCT levels and correlates assessed by same rater-population) versus between-context ratings (SCT levels assessed based on ratings of observer population A but correlates of SCT rated by observer population B).

With regard to other socio-demographic variables, it has been observed that gender ratios differed according to ADHD subtype and SCT diagnosis [30]. Todd and colleagues [30] showed that SCT item loadings were different for males and females. Accordingly, two SCT items (“day dreams”, “low energy”) were identified to form a separate factor in boys (explaining 6.6 % of the total variance) but not in girls. In girls, the two SCT items (“day dreams”, “low energy”) loaded on the inattentive factor of ADHD/I. The authors concluded that boys with ADHD and SCT are best described by an inattentive and a hyperactive-impulsive factor, whereas girls’ subtyping was not facilitated by the inclusion of SCT symptoms [30]. However, Garner and colleagues [13] showed that SCT symptoms were generally increased in boys prone to behavioral deviance and/or individuals with an ADHD/I diagnosis. Various research on ADHD shows that ADHD is more closely linked with being male than female. Male-to-female ratios of ADHD diagnosed individuals have been shown to range from 9:1 to 6:1 [52], with community-based samples presenting with a ratio of approximately 3:1 [52]. Research on gender differences in ADHD is, however, highly needed [53]. Not only should attention be drawn on methodological limitations such as gender-biased diagnostic tools, but also on the possibility of confounding effects due to referral biases [53]. For example, girls from community-based samples of ADHD presented on average with lower levels of inattention, internalizing behavior and peer aggression than boys with ADHD, whereas clinical samples of ADHD did not show any differences on these variables for gender [53]. It should be questioned whether differences in gender on SCT and ADHD are a mere effect of referral-bias or whether future research should handle different standards in evaluating SCT and ADHD in line with the individual’s gender.

With regard to mean age of symptom-onset of SCT, no differences were found between ADHD samples with low SCT and high SCT [27]. Bauermeister and colleagues [12], however, found a later onset of inattention and SCT symptoms in their group of individuals with inattention. However, it remains unclear whether these differences in mean onset age of symptoms are indeed due to differences in samples or simply due to a distortion associated with retrospective assessments of ADHD/SCT symptoms. Moreover, Bauermeister and colleagues’ sample [12] was characterized by a low impairment in adaptive functioning and the assessed mothers reported only little to no child-related family stress. The results of Harrington and Waldman [27], in contrast, stem from clinically referred children who were seen due to their suspiciousness for attentional and behavioral deviances. It can be speculated that the onset time of symptoms is either better remembered for those who are suspicious of severe behavioral deviance [27] compared to those who are rather adapted [12] or that the onset of symptoms indeed differs due to differences in symptom severity.

### SCT and social functioning

Within the last decade the interest in ADHD diagnosed individual’s social and emotional functioning is on the increase [54–57]. It was already mentioned above that individuals with SCT are more prone to internalizing behaviors [2, 5, 8, 11, 13, 17, 37, 51], such as turning inward, not expressing certain needs, and appearing rather shy. Internalizing behaviors have been further shown to increase the proneness of being less socially interactive. It can be assumed that a decreased opportunity for social interactions might affect social functioning in general and vice versa. In line with this, it was shown that children with ADHD/I with SCT tended to take less initiative in social situations and were rated to be less assertive and more self-controlled during home and school based observations [12]. This finding was not replicated in a later study in which inappropriate on-task behavior was found to be unrelated to SCT but associated with symptoms of inattention [11]. While both studies [11, 12] used information obtained from behavioral observations during task performance [12] or class-room performance [11] to measure social functioning, the authors failed to implement a standardized measure of social functioning.

Mikami and colleagues [23] in contrast applied computer simulated peer interaction in order to measure social skills of children with ADHD and SCT compared to different ADHD subtypes. In line with the observation that children with ADHD/I and SCT have more social problems, are more likely to be socially withdrawn [17, 51], less happy and more anxious in social interactions [17], Mikami and colleagues [23] found fewer responses, a weaker memory and a reduced ability to attend to subtle social cues in children with ADHD/I and SCT compared to children with ADHD/I without SCT. Moreover, a relation between SCT and children’s hostility was observed [23] with children suffering from ADHD/I and SCT showing less symptoms of hostility than the group of children with ADHD/I but without SCT [23]. A reduced hostility of children with SCT was further confirmed by Becker and colleagues [5], supporting the formerly proposed positive impact of SCT on severe disruptive behavior [17, 20, 23]. Again, it can be speculated that even though SCT seems to impact negatively on the individuals social functioning, by making individuals more socially reluctant and less attentive to social cues [23], SCT also functions as a protective factor, making the individual more resilient to deviant behavior such as hostility [5, 23].

Another consequence of impaired social functioning associated with SCT might be seen in increased levels of peer-rejection. For example, teachers’ ratings of children on the item “cannot pay attention and looks sleepy” were related to greater peer rejections in pupils independent of internalizing, anxious or depressed features of the assessed child [49]. The authors concluded that SCT was the only predictive factor with regard to peer rejection [49].

So far, no study focused on social perceptual functioning in children with SCT. Future studies should therefore implement the measurement of social cognitive as well as social perceptual performances of children with SCT in order to bridge the gap between SCT and social-perception as well as social-cognition. The observation that social deficits might arise due to slower responses or inattention to meaningful social cues might further plead for research assessing the timing sensitivity of children with SCT in social situations. In particular, considering that children with ADHD have a poorer perception of time [20, 58] and timing functions have been shown to be crucial in social interactions [59, 60], the examination of a relationship between abnormal timing functions and impaired social functioning in children with SCT could enhance the understanding of social shortcomings seen in SCT.

### SCT treatment efficacy

Clinical observations suggested that treating children with ADHD/I and ADHD/C together might be detrimental for children with ADHD/I [61], underlining hereby the need for individualized treatments according to patients’ unique psychopathology. Similar, children with ADHD/I and SCT have been shown to benefit the most from treatment that addresses the processing deficits and social impairments associated with ADHD/I [32]. For example, SCT symptoms were shown to be as responsive as ADHD/I symptoms to the Child Life and Attention Skill (CLAS) program devised by Pfiffner and colleagues [32]. Furthermore, the positive effects remained stable at follow-up [32]. The CLAS program was adapted from a program for the treatment of mild closed head injury in children and is characterized by prompts, routinization and task complexity reduction. Unfortunately, no control group was examined in this study so that it remains unclear to what amount the observed improvements of children were caused by nonspecific effects of treatment, such as the positive influence of the teacher or parent training. Ludwig and colleagues [62] showed that individuals with ADHD/I and SCT do not differ in their response to stimulant drug treatment using methylphenidate from individuals with ADHD/I without SCT. The CLAS program [32] might, therefore, be a promising approach to address symptoms seen in children with ADHD/I and SCT.

## Conclusion

The current review’s aim was to provide a comprehensive overview of the psychometric as well as empirical validity of SCT, its etiology, its unique contribution to individuals’ neuro-cognitive profiles, its impact on individuals’ social and emotional well-being, as well as its treatment. Even though there seems to be no consensus yet whether SCT could account as a disorder itself [11, 16], recent studies confirm the link between ADHD/I and SCT [1, 10, 13, 23, 29, 36, 38].

The removal of SCT symptoms from the DSM-IV criteria of ADHD resulted in a loss of relevant information about cognitive impairments associated with ADHD/I [41] and might even lead to overdiagnosing of ADHD/C, in particular of those cases that would rather fit the category of ADHD/I and SCT or pure SCT [41]. The fact that the items assessing SCT were not reintroduced into the current DSM-5 might increase ADHD’s homogeneity artificially [14, 26], leading thereby to a distorted diagnosis and treatment of individuals with ADHD.

Furthermore, SCT was present in or comorbid to a variety of clinical or physical disorders different from ADHD (e.g., clinical-referred children [13]; FAS [3]; leukemia survivors [4]), increasing the need for future research on SCT in a variety of clinical populations. The current review points out that it is warranted to assess for SCT symptoms in patients suffering from attention deficits within the context of neuro-cognitive treatment and rehabilitation.

However, before SCT can be used in research or clinical settings, its construct and empirical validity needs to be substantiated. Based on the research studies performed in the field of SCT, the current review concludes that SCT measures vary widely across studies and lack standardization. Three components of SCT (i.e., “slow”, “sleepy”, “daydreamer” [2]) however, seem promising for being implemented in future studies on SCT. By applying these unique SCT items, SCT emerged as a psychometrically valid factor which is even distinct from ADHD/I [2, 14]. Moreover, each of these three components was found to be independently related with different comorbidities [2]. Accordingly, whereas the “slow” component was foremost related to ADHD/I, ADHD in general, and ODD, the other two components “sleepy/daydreamer” were more likely to be related to a more pure form of SCT [2]. Even though some of the studies tried to integrate the individual impact of each of these three components of SCT [11, 13, 24], none of the studies explicitly assessed the items’ unique associations with ADHD subtypes or other comorbidities. The aim of future research could be to take the mentioned components as a starting point for further analysis of SCT and its behavioral as well as cognitive markers.

The majority of the reviewed studies were based on teacher and parent ratings. Self-measurements and objective measures of SCT (e.g., computerized cognitive tasks) are lacking so far, however, it was proposed that the nature of SCT symptoms requires longer behavioral as well as cognitive observations. Simple observations of ten minutes of on-task behavior were, for example, considered to be insufficient to reasonably capture SCT [29].

Whereas a link between sustained attention deficits and SCT was consistently found [24], the hypothesized association of SCT with both cognitive and behavioral speed [19, 58] or slowed information processing in general [12, 14, 17] was rather inconsistent and needs further investigation. It has been hypothesized that a decreased processing speed might lead to a general distortion in individuals’ perception of time [58] and impacts on several timing functions such as motor- and perceptual timing, as well as temporal foresight [63]. These timing functions, however, are important for motor control, decision making and the individual’s psychological orientation in time [64]. Several studies so far showed that patients with ADHD present with a different sense of time [58, 65, 66], see [63] for a recent review. Given SCT’s strong link to ADHD and its proposed association with slowed processing speed, it would be interesting to examine the impact of SCT on timing functions in general and perceptual timing in particular.

The findings concerning the impact of SCT on classroom behavior and children’s academic achievement remain inconsistent [10, 36], however, SCT’s impact on children’s social- as well as emotional well-being has been shown to be concerning [5, 37, 51], with SCT showing a clear link to internalizing disorders such as anxiety and depression [2, 8, 11, 13, 37].

While the majority of studies focused on adolescents or children, only one study examined SCT in adulthood [16]. Because of the clear association between ADHD and SCT and the fact that ADHD persists from childhood throughout adulthood [67], thorough examinations of SCT in longitudinal studies would be desirable. These studies could, for instance, provide information about the presence and consequences of SCT in community-based as well as clinical populations and add valuable insights into the developmental trajectory of SCT.

The only genetic study available in this field indicates that SCT seems to share some of the genes with ADHD but is most strongly associated with environmental factors [26]. The idea, that SCT is a by-product of ADHD related environmental factors [26] seems worth to be further studied in future research. Based on Barkley’s [16] classification of SCT symptoms, it can be assumed that if a threshold of 5 or more out of 9 SCT symptoms will be applied as a standard for diagnosing SCT, 5.1% of the general population would be diagnosed with SCT, which is comparable to the number of ADHD diagnoses in children [52]. Barkley’s estimate therefore gives an impression of the number of patients which can be expected if SCT establishes as a disorder itself.

Based on the discussed literature, it can be concluded that SCT in ADHD but also SCT in other populations and pathologies is understudied. Whereas first attempts for valid and consistent measurements have been made [2], recent studies on SCT lack coherence and standardization of measurement. To allow the diagnosis of SCT, a consensus about which dimensions actually represent SCT has to be reached first. Two factors, represented by the condition “sleepy/daydreamer” seem promising for a future taxonomy of SCT [2]. Neurocognitive tests focusing on timing functions and attention (in particular sustained attention) should be combined with behavioral observations that target sleepy/daydreaming behavior and absent-mindedness, without missing the importance of assessing SCT’s link to internalizing and other mood disorders. Especially, patients with depressive disorders or acquired brain lesions (e.g. during the phase of neuro-rehabilitation) might benefit from a thorough assessment of comorbid SCT. SCT symptoms have been shown to improve by means of non-pharmacological treatment [32]. As these results are rather preliminary, additional psycho-educative, therapeutic, and behavioral interventions should be tested for their efficacy in populations with clinical levels of SCT. Studies on the genetic background of SCT should be encouraged, testing hereby the assumption whether SCT is a by-product elicited by the environmental constraints put forward by the disorder to which SCT is comorbid to (e.g., ADHD).

## Electronic supplementary material

Additional file 1: Table S1: Studies and measures employed [68–162]. (DOCX 86 KB)
