# Supplementary material for: Sluggish cognitive tempo and its neurocognitive, social and emotive correlates: a systematic review of the current literature
Source: J Mol Psychiatry. 2014 Aug 5;2:5. doi: 10.1186/2049-9256-2-5 (PMC4416322; doi:10.1186/2049-9256-2-5)
Supplement: Supplementary file 1 — Additional file 1: Table S1: Studies and measures employed [68–162]. (DOCX 86 KB) [file 40303_2014_23_MOESM1_ESM.docx]

Additional file 1: Table S1. *Studies and measures employed*

| **Author(s)** | **Population** | **Medication Status** | **Sample(s) ^a^** | **SCT Measure(s)^b^** | **Other Measure(s)** |
| --- | --- | --- | --- | --- | --- |
| Lahey et al., 1987 [44] | **C** | **MU** | **Evaluated population:**  63 children with ADHD  Age range: 9-13 yrs.  (51 males; 12 females)  (a) ADHD (n=41)  (b) ADD (n=22)  **Raters:**  Teachers  Parents  Researchers | **SCT scale from Schedule for Affective Disorders and Schizophrenia for School-Age Children** (K-SADS: [68]) | **Schedule for Affective Disorders and Schizophrenia for School-Age Children** (K-SADS: [68])**:** ADHD  **Conners’ Parent Rating Scale** **Revised Behavior** [70]: ADHD, psychopathology  **Revised Behavior Problem Checklist** [70]: Behavioral functioning |
| Schaughency et al., 1992 [49] | **CB** | **NA** | **Evaluated population:**  110 children  Age range: 7-11 yrs.  (42 males; 68 females)  **Raters:**  Teachers  Classmates | **Comprehensive Behavior Rating Scale for Children** (CBRSC:[44]): “Looks sleepy” in peer ratings “Sluggish tempo” in teacher ratings. | **“Likes best/Likes least” Ratings** [71] |
| Skansgaard et al., 1998 [29] | **CB** | **NA** | **Evaluated population:**  24 children  Age range: 7-11 yrs.  (a) ADHD/C (n=6)  (b) ADHD/I (n=6)  (c) Controls (n=12)  **Raters:**  Teachers  Researchers | **2 items SCT Scale from Child and Adolescent Disruptive Behavior Inventory: Teacher Rating Scale 2 (**CADBI-TRS2: [72])**:** “Daydreams (e.g., stares into space) in class”, “Is low in energy, sluggish or drowsy”  **4 items SCT Scale from Direct Observation Form** (DOF: **[**50])**:** ”Confused or seems to be in a fog”, ”Daydreams or gets lost in thoughts”, “Stares blankly”, “Underactive, slow moving, lacks energy, or yawns” | **Child and Adolescent Disruptive Behavior Inventory: Teacher Rating Scale 2** (CADBI-TRS2: [74])  **DOF** [50,75]**:** behavioral functioning |
| McBurnett, Pfiffner & Frick, 2001 [19] | **C** | **MU** | **Evaluated population:**  692 children and adolescents with ADHD  Age range: 3-18 yrs.  (546 males; 146 females)  **Raters:**  Teachers  Parents | **SCT items from DSM-IV symptom list:** “Daydreams a lot”, “Often is sluggish or drowsy” | **Revised Swanson, Nolan and Pelham Questionnaire** (SNAP-R: [74])**:** psychopathology according to DSM-IV |
| Carlson et al., 2002 [17] | **CB** | **NA** | **Evaluated population:**  346 children  Age range: 4-11 yrs.  (180 males; 166 (females)  (a) ADHD/C (n=50)  (b) ADHD/I high SCT (n=34)  (c) ADHD/I low SCT (n=89)  (d) Controls (n=123)  **Raters:**  Teachers | **5-item SCT Scale:** Five items from the Child Behavior Checklist-Teacher Rating Form (CBCL-TRF: [35,14]): “Sluggish/slow to respond”, “Seems to be in a fog”, “Drowsy or sleepy”, “Easily confused”, and “Daydreams/stares into space” | **DSM-4 checklist for ADHD, ODD** [53]  **3 Item-Social functioning scale** [75]  **CBCL-TRF** [35]: Childhood behavior |
| Weiler et al., 2002 [76] | **C** | **OFF** | **Evaluated population:**  230 children with ADHD  Age range: 7.6-11.1 yrs.  (111 males; 119 females)  (a) ADHD (n=24)  (b) Reading disability (RD)  (n= 42)  (c) Comorbid ADHD and RD (n=9)  (d) Controls (n=149)  **Raters:**  Teachers | **Class-room observations** [76] | **Kaufman Brief Intelligence Test** (K-BIT: [77)]: IQ  **Visual Filtering Task** [76]**:** Parallel search, Serial search, Decision and Motor response  **Rapid Auditory Processing Task** [78]: Auditory Processing, Decision and Motor response |
| Hartman et al., 2004 [14] | **C** | **MW** | **Evaluated Population:**  296 twins with ADHD and learning disabilities  Age range: 8-18 yrs.  (141 males; 145 females)  **Raters:**  Teacher  Parents | **5 SCT items** based on [e.g., 9,35]: “Sluggish/slow to respond”, “Seems to be in a fog”,” Drowsy or sleepy”, “Easily confused”, and “Daydreams/stares into space” | **Disruptive Behavior Rating Scale** (DBRS: [35]): ADHD  **DSM-III-R parent-report version of the Diagnostic**  **Interview for Children and Adolescents** (DICA-P: [80]): ODD, conduct disorder (CD), generalized anxiety disorder (GAD), and major depressive disorder  **Child Behavior Checklist** (CBCL: [35]): Internalizing/Externalizing behavior  **Wechsler Intelligence Scale for Children** (WISC-R: [81]): IQ  **Wechsler Adult Intelligence Scale** (WAIS: [82]): IQ  **Peabody Individual Achievement Test** (PIAT: [83]): Academic achievement |
| Todd et al., 2004 [30] | **CB** | **NA** | **Evaluated Population:**  229 twins  Age range: 8-14 yrs.  (109 males; 120 females)  (a) No ADHD (n=163)  (b) ADHD/I (n=30)  (c) ADHD/C (n=29)  (d) ADHD/HI (n=7)  **Raters:**  Parents | **2 SCT-items:** “Stares into space and daydreams”, “Appears to be low in energy, sluggish, or drowsy” | **DSM-IV:** ADHD and questions about major depression (MDD), generalized anxiety disorder (GAD), social phobia, and other disorders |
| Bauermeister et al., 2005 [12] | **CB** | **NA** | **Evaluated population:**  98 children,  Age range: 6 - 11 yrs.  (58 males; 40 females)  (a) Inattentive and hyperactive group (n=47)  (b) Inattentive only  (n=44)  (c) Control (n=29)  **Raters:**  Teachers  Mothers | **5-item SCT scale** (SCT-5)**:** “Confused or seems to be in a fog”, “Daydreams or gets lost in his/her thoughts”, “Stares blankly”, “Underactive, slow moving or lacks energy”, and “Apathetic or unmotivated” | **School Behavior Inventory** (SBI: [84]): Distraction-Motivation (DM) and Activity-Impulsivity (AI) scales  **Disruptive Behavior Rating Scale** (DBRS:[ 58]): Behavior functioning  **Child Behavior Checklist** (CBCL; [73]):  Behavioral functioning  **Developmental and Diagnostic Interview DISC-IV** [85]: DSM-IV psychopathology  **Children’ s Global Assessment Scale**-**Spanish** (CGAS:[86]): Adaptive functioning  **Home and School Situations Questionnaires** (HSQ & SSQ: [58]): Behavior functioning  **It’s About Time Questionnaire** (IATQ: [58]): Sense of time  **Spanish Social Skills Questionnaire** (SSSQ: [87]): Social functioning  **Wechsler Intelligence Scale for** **Children–Revised for Puerto Rico** (WISC-R PR: [88]): IQ  **Spanish Spelling-Test of Reading and Written Language** (TRWL: [89]): IQ  **Bender-Gestalt Test** (BGT: [90]: Visual-motor integration  **Conners’ Continuous Performance Test** (CPT: [91]): Vigilance, impulsiveness  **Restricted Academic Situation Behavior Coding System** [15]: Behavioral functioning  **Solid State Actigraph** [92]: Number of movements a child makes per unit of time  **Beck Depression Inventory–Spanish** (BDI: [93]): Mother self-report measure  **Family APGAR–Spanish** [94]: Parent’s satisfaction with family relationships  **ADHD Symptoms Scale – Self-Report Form** (ADHD SS-SRF: [58]): ADHD  **Family Experiences Inventory** (FEI: [95]): Family stress  **Parent Practices Inventory** (PPI: [96]): Parental monitoring and supervision, parental involvement, parental discipline |
| Huang-Pollock et al., 2005 [41] | **C** | **MW** | **Evaluated population:**  79 children  Age range: 8–12 yrs.  (a) ADHD/I (n=16)  (b) ADHD/C (n=28)  (c) Control (n=35)  **Raters:**  Teachers  Parents | **3-item SCT scale** [17,19]: “Stares into space/ daydreams”, “Low in energy, sluggish, or drowsy”, “Apathetic or unmotivated to engage in goal-directed activities” | **Behavior Assessment Scale for Children** (BASC: [97]): Attention, hyperactive, or conduct problem scale  **ADHD rating scale** [98]: ADHD  **Conners’ Rating Scales- Revised** [99]: ADHD  **Developmental and Diagnostic Interview DISC-IV** [85]: DSM-IV psychopathology  **Selective attention paradigm** [101,102]: Selective attention |
| Hinshaw et al., 2007 [34] | **C** | **MW** | **Evaluated population:**  228 girls  Age range: 6-12 yrs.  (a) Psychiatrically-referred girls (n=140)  (b) Controls (n=88)  **Raters:**  Researchers | Group of ADHD with SCT was formed based on: (a) presence of very few HI symptoms, (b) high levels of parent- and teacher- rated sluggish cognitive tempo | **Wechsler Intelligence Scale for Children** (WISC-III: **[**102]): IQ  **Taylor Complex Figure Test** (TCFT: [103]): Planning, perceptual organization, graphomotor abilities  **Conners’ CPT** [91]: Visual attention, set shifting  **Rapid Automatized Naming Test** (RAN: (150)]: Orienting, semantic processing and retrieval  **Underlining Test** [104,105]: Word recognition, controlled processing |
| Mikami et al., 2007 [23] | **C** | **MW** | **Evaluated population:**  116 children  Age range: 7-12 yrs.  (81 males; 35 females)  (a) ADHD-C (n=33)  (b) ADHD-I (n=45)  (c) Control (n=38)  **Raters:**  Teachers  Parents | **SCT items from DSM-IV scale:** “Often daydreams” , “Sluggish or drowsy”, “Apathetic and unmotivated” | **Cooperation subscale of the Social Skills Rating System** (SSRS: [87]): Social functioning  **Peer Relations Scale** [112]: Peer-ratings: subscales of “positive nominations” (measuring whether the child is sought out and liked by peers), and “negative nominations” (measuring whether the child is actively disliked by peers)  **Typing ability**: Time to accomplish 3 standard sentences  **Wechsler Intelligence Scale for Children**, 4th ed (WISC-IV: [106]), or **Wechsler Abbreviated Scale of Intelligence** (WASI: [107]): IQ  **Word Reading and Reading Comprehension subtests on the Wechsler Individualized Achievement Test, 2nd ed** (WIAT-II: [108]): Reading ability  **Computerized Chat-Room Peer Interaction:** Social functioning |
| Pfiffner et al., 2007 [32] | **C** | **OFF** | **Evaluated population:**  69 children with ADHD/I symptoms  Age range: 7-11yrs.  (46 males; 23 females)  **Raters:**  Teachers  Parents | **15-item SCT Scale** (SCT-15:[109]) | **DSM-IV Inattention Symptoms** [110]**:** Inattention  **Social Skills Rating System (**SSRS: [87]): Social functioning  **Children`s Organizational Scale** (COSS: [111]): Organizational functional  **Test of Life Skill Knowledge** [112]**:** Social and organizational functioning  **Clinical Global Impressions Improvement** [113]: Psychopathology |
| Reeves et al., 2007 [4] | **C** | **OFF** | **Evaluated population:**  99 children and adolescents  Age range: 6-19 yrs.  (53 males; 46 females)  (a) Survivors of acute lymphoblastic leukemia (n=80)  (b) Controls (n=19)  **Raters:**  Researchers | **SCT** **scale (SCT-5) of Child Behavior Checklist** (CBCL: [35]): ‘‘Confused or in a fog’’, ‘‘Daydreams or gets lost in his or her thoughts’’, ‘‘Overtired’’, ‘‘Stares blankly’’, ‘‘Underactive, slow moving, or lacks energy’’ | **Wechsler Intelligence Scale for Children**, third edition (WISC-III: [102]): IQ  **Wechsler Adult Intelligence Scale** [114]: IQ  **Wechsler Individual Achievement Test** (WIAT: [114]): Academic functioning |
| Solanto et al., 2007 [31] | **CB** | **NA** | **Evaluated population:**  80 children  Age range: 7-11 yrs.  (43 males; 37 females)  (a) ADHD/I (n=26)  (b) ADHD/C (n=34)  (c) No ADHD but other psychiatric condition (n=20)  **Raters:**  Teachers  Parents | **2 Items of SNAP-IV Teacher and Parent Rating Scale** [116]**:** “Stares into space and reports daydreaming”, “Appears to be low in energy levels, sluggish or drowsy” | **Delay Aversion Test** (DAT: [117]): Delay aversion  **Buschke Selective Reminding Test** [118]: Verbal learning and memory  **Visual Learning Test of the WRAML** (VLT: [119]): Spatial memory  **Sternberg visual memory search task** [120]: Information-processing  **Stroop Color-Word Test** [121]: Cognitive flexibility, resistance to interference  **Conners’ Continuous Performance Test** (CPT: [40]): Inhibitory control  **Posner Test** [122]: Covert visuospatial orienting task  **Tower of London** (TOL: [123]): Planning  **Stimulus–Response Processing Task** (SRPT: [31]): Target detection  **Wisconsin Card Sorting Test** (WCST: [124]): Cognitive set shifting, inhibition  **Warned Simple Reaction Time Test** (WSRT: [31]): Reaction time |
| Desman et al., 2008 [38] | **C** | **ON** | **Evaluated population:**  38 boys  Age range: 8–12 yrs.  (a) ADHD/I (n=19)  (b) Controls (n=19)  **Raters:**  Researchers | Children with ADHD/I that responded slower on Go/No-Go paradigm in line with SCT symptomatology | **DSM-IV** [7]: ADHD  **Conner’s Parent Rating Scale** (CPRS 10-item index: [69]): Psychopathology  **Strength and Difficulties Questionnaire** (SDQ-G: [125]): Hyperactivity  **The Go/No-Go paradigm of the Test Battery for Attentional Performance** (TAP: [126,127]): Inhibitory control, interference |
| Ludwig et al., 2009 [62] | **C** | **ON** | **Evaluated population:**  88 ADHD/I children and adolescents  Age range: 8-15 yrs.  (63 males; 25 females)  (a) ADHD/I and SCT (n=18)  (b) ADHD/I no SCT (n=70)  **Raters:**  Researchers | **SCT scale from Child Behavior Checklist** (CBCL: [35]): ‘‘Being confused or lost’’ , ‘‘Daydreaming’’, ‘‘Stares’’, ‘‘Drowsiness’’. | **Kiddie Schedule for Affective Disorders and Schizophrenia–Epidemiological version** (K-SADS-E: [128,129]): Psychopathology  **Swanson, Nolan, and Pelham Questionnaire**  **scale–version IV** (SNAP-IV: [130]): ADHD  **Wechsler Intelligence Scale–Third edition**  (WISC-III: [102]): IQ |
| McConaughy et al., 2009 [50] | **C** | **OFF** | **Evaluated population:**  136 children  Age range: 6-11 yrs.  (116 males; 47 females)  (a) ADHD/C (n*=* 64)  (b) ADHD/I (n*=* 22)  (c) Psychiatrically-referred children without ADHD (n= 51)  (d) Controls (n=26)  **Raters:**  Teachers  Parents | **SCT scale of Direct Observation Form** (DOF: [29,50]) | **ADHDRS-IV** [98]: ADHS  **NIMH DISC-4** [85]: Psychopathology  **DOF** [50]: Standardized form for rating observations of children’s behavior in classrooms, at recess, and in other group settings. |
| Penny et al., 2009 [2] | **CB** | **NA** | **Evaluated population:**  335 children  Age range: 4-13 yrs.  **Raters:**  Teachers  Parents | **14 Item SCT scale** [2]: “Appears to be sluggish”, “Is apathetic: shows little interest in things or activities”, “Appears tired, lethargic”, “Seems to be in a world of his or her own” , “Seems drowsy”, “Daydreams”, “Gets lost in his or her own thoughts”, “Is unmotivated”, “Underactive, slow moving, or lacks energy”, “Needs extra time for assignments”, “Lacks initiative to complete work”, “Effort fades quickly”, “Yawning, stretching, sleepy-eyed appearance”, “Slow or delayed tasks” | **Disruptive Behavior Disorder rating scale** (DBD: [131]): ADHD, oppositional defiant disorder (ODD), conduct disorder (CD)  **Internalizing subscale of the Pediatric Symptom Checklist** (PSC: [132]): Internalizing |
| Garner et al., 2010 [13] | **C** | **MU** | **Evaluated population:**  322 Psychiatrically-referred children and adolescents  Age range: 5-17 yrs.  (a) ADHD-I (n=40)  (b) ADHD-C/H (n=90)  (c) Learning Disability or Language Disorder (n=32)  (d) Controls (n=44)  **Raters:**  Teachers  Parents | **5-item SCT scale (SCT-5) from the Child Behavior Checklist** (CBCL: [35]): “Sluggish/slow to respond, ”Seems to be in a fog,” , “Drowsy or sleepy”, “Easily confused”, and “Daydreams/stares into space” | ***DSM-III-R* parent-report version of the Diagnostic Interview for Children and Adolescents** (DICA-P: [80]): symptoms of oppositional defiant disorder (ODD), conduct disorder (CD), generalized anxiety disorder (GAD), and major depressive disorder.  **Child Behavior Checklist** (CBCL: [35]): Behavioral functioning  **Wechsler Intelligence Scale for Children** (WISC-R: [81]): IQ  **Wechsler Adult Intelligence Scale** (WAIS:[ 82])**:** IQ  **Peabody Individual Achievement Test** (PIAT: **[**83]): Reading and mathematics |
| Wåhlstedt et al., 2010 [24] | **CB** | **NA** | **Evaluated Population:**  209 children  Age range: 8-9 yrs.  (111 males; 98 females)  **Raters:**  Teachers  Parents | **5 SCT** **items from Child Behavior Checklist** (CBCL: [35])**:** “Confused or seems to be in a fog”, “Daydreams or gets lost in his/her thoughts”, “Stares blankly”, “Underactive, slow moving or lacks energy”, “Apathetic or unmotivated” | **Stroop-like day-night test** [134,135]: Inhibitory control  **Children’s Size-Ordering Task** [136]: Working memory  **Pig House:** Spatial working memory task  **Go-/No-go** [137,138,139]: Sustained attention, state regulation  **Wechsler Intelligence Scale for Children – third edition** (WISC-III:[102]): IQ  **DSM-IV** [7]: ADHD, ODD  **Strengths and Difficulties Questionnaire** (SDQ: [139]): Internalizing symptoms  **Academic achievement:** Swedish, mathematics, and social sciences |
| Skirbekk et al., 2011 [33] | **C** | **OFF** | **Evaluated population:**  141 children  Age range: 7-13 yrs.  (90 males; 51 females)  (a) ADHD and Anxiety disorder (n=25)  (b) ADHD (n=39)  (c) Anxiety disorder (n=41)  (d) Controls (n=36)  **Raters:**  Researchers  Mothers | **17-item SCT scale** (SCT-17: [32]): “Daydreams”, “Stares into space”, “In a fog”, “Unresponsive”, “Easily confused”, “Mind wanders”, “Absentminded”, “Easily disoriented”, “Loses cognitive set”, “Gets tongue tied”, “Poor memory consolidation”, “Drowsy”, “Moves slowly”, “Works slowly and takes a long time to complete tasks”, “Gets tired easily”, “Low initiative”, “Poor time appreciation”.  **5-item SCT scale from the Child Behavior Checklist** [CBCL: 18,37]: “Sluggish/slow to respond”, “Seems to be in a fog”, “Drowsy or sleepy”, “Easily confused”, “Daydreams/stares into space” | **Children’s Global Assessment Scale** (CGAS:[143]): Child’s overall severity of disturbance  **Disruptive Behavior Rating Scale** (DBRS:[144]): ADHD  **Wechsler Abbreviated Scale of Intelligence** (WASI: [107]): IQ  **Attention Network Test** (ANT; [142]): Reaction time  **Digit Span subtest of the WISC-III** [102]: Verbal memory span, working memory  **Adaptation of the Finger Windows subtest from the Wide Range Assessment of Memory and Learning** (WRAML: [119]): Spatial memory |
| Barkley, 2012 [16] | **CB** | **NA** | **Evaluated population:**  1.249 adults  Age range: 18-96 yrs.  (a) No ADHD but SCT (n=33)  (b) ADHD but no SCT (n=46)  (c) ADHD and SCT (n=39)  (d) Controls (n=1131)  **Raters:**  Adults themselves | **Adult SCT rating** [143]:  **9 items:** “Prone to daydreaming when I should be concentrating”, “Have trouble staying alert or awake in boring situations”, “Easily confused”, “Easily bored”, “Spacey or in a fog”, “Lethargic, more tired than others”, “Underactive or have less energy than others”, “Slow moving”, “I don’t seem to process information as quickly or as accurately as others” | **Adult ADHD Rating Scale-IV** [143]: ADHD  **Functional Impairment Rating Scale** [144]: Impairment in 15 major life domains  **The Deficits in Executive Functioning Scale** [145]: Executive functioning |
| Bauermeister et al., 2012 [11] | **CB** | **NA** | **Evaluated population:**  140 children  Age range 6-11 yrs.  (87 males; 53 females)  **Raters:**  Teachers  Researchers | **4 Sluggish items from Child Behavior Checklist** (CBCL: [35]): “Confused or seems to be in a fog”, “Daydreams or gets lost in his/her thoughts”, “Stares blankly” and “Underactive, slow moving, or lacks energy” | **ADHD Scales of the Disruptive Behavior Rating Scale** [162]**:** ADHD  **Observed Behavior- Continuous Performance Test** (CPT: [91]): Vigilance, impulsiveness  **Hand Movements Scale** (HMS: [146]): Skilled hand movement, nonverbal working memory, motor sequencing  **Simon Task** (ST: [147])**:** Nonverbal working memory  **Stroop Color-Word Test** (SCWT: [121]): Interference control  **Wisconsin Selective Reminding Test** (WSRT: [148]): Verbal learning and memory test adapted from Buschke’s Selective Reminding Test [149]  **WISC-R PR Backward Digit Span Test** [88]: Verbal working memory  **Rapid Automatized Naming** (RAN: [150]): Digit-, color-, object-naming  **Woodcock Psychoeducational Battery-Spanish** (WPB-S: [151]): Academic functioning  **Child Behavior Checklist (CBCL) and CBCL-Teacher Report Form (TRF)** [35]: Internalizing, externalizing, social problems, thought problems, and attention problems  **Social Skills Questionnaire**  [87]: Cooperation, assertion, self-control scales, parent responsibility scale |
| Harrington et al., 2012 [27] | **C** | **MU** | **Evaluated population:**  291 children  Age range: 5-18 yrs.  (206 males; 85 females)  (a) ADHD/C (n=124)  (b) ADHD/H (n= 23)  (c) ADHD/I (n=81)  (d) Controls (n=63)  **Raters:**  Mothers | **SCT items from Emory Combined Rating Scale** (ECRS: [152]): “Forgetful”, “Daydreams”, “Sluggish/drowsy” | **The Emory Combined Rating Scale** (ECRS: [152]): Psychopathology |
| Jacobson et al., 2012 [36] | **C** | **ON** | **Evaluated population:**  143 children (no formal diagnosis of ADHD but high on symptoms of ADHD)  Age range: 3-18 yrs.  (96 males; 47 females)  (a) Low ADHD symptoms (n=85)  (b) High ADHD/I (n=37)  (c) High ADHD/HI (n=8)  (d) High ADHD/C (n=13)  **Raters:**  Teachers | **SCT 14-item scale** [2] | **ADHD Rating Scale-IV** [98]: ADHD  **Vanderbilt Assessment Scales** [153]: Internalizing    **Impairment Rating Scale** [154]: Behavioral functioning |
| Becker et al., 2013 [5] | **C** | **MU** | **Evaluated population:**  680 children  Age range: 6-12yrs.  (497 males; 183 females)  (a) ADHD only (n=102)  (b) ODD only (n=61)  (c) CD only (n=41)  (d) Anxiety disorder only (n=54)  (e) Mood disorder only (n=41)  (f) ADHD+ODD (n=190)  (g) ADHD+CD (n=34)  (h) ADHD+anxiety disorder (n=82)  (i) ADHD+mood disorder (n=75)  **Raters:**  Parents | **5 SCT items from CBCL 6-18** (SCT-5: [155]): “Sluggish/slow to respond”, “Seems to be in a fog”, “Drowsy or sleepy”, “Easily confused”, and “Daydreams/stares into space” | **CBCL** [155]: Mental health, social functioning, ADHD, SCT, anxiety, depression, ODD  **Observed Behavioral Dysregulation:** Assessment form of behavior modification program    **Hopkins Symptom Checklist** (HSCL: [156]): Parent anxiety and parent depression symptoms |
| Becker & Langberg, 2013 [37] | **C** | **MU** | **Evaluated population:**  52 adolescents  Age range: 12–16 yrs.  (37 males; 15 females)  (a) ADHD/I (n=27)  (b) ADHD/C (n=25)  **Raters:**  Teachers  Parents | **SCT-14 item scale** [2] | **Wechsler Intelligence Scale for Children—4^th^ Edition** (WISC-IV: [106]): IQ  **Wechsler Individual Achievement Test, 3^rd^ Edition** (WIAT-II: [106]): Academic achievement  **Vanderbilt ADHD Diagnostic Rating Scale** (VADRS: **[**153]): ADHD  **Behavior Rating Inventory of Executive Function** (BRIEF: [157]): Behavioral functioning |
| Graham et al., 2013 [3] | **C** | **MU** | **Evaluated population:**  272 clinically referred children  Age range: 8- 16 yrs.  (162 males; 110 females)  (a) alcohol-exposed children with ADHD (n=75),  (b) alcohol-exposed children without ADHD (n=35),  (c) nonexposed children with ADHD (n=60),  (d) Controls (n=102)  **Raters:**  Parents | **SCT Scale** (SCT-S: [19,112]) | **Child Behavior Checklist** (CBCL: [35]): Behavioral functioning  **Computerized Diagnostic Interview Schedule for Children—Fourth Edition** (C-DISC-4.0: [85]): ADHD  **Dysmorphology examination** (for details, see [158,159]) |
| Langberg et al., 2013 [10] | **C** | **MU** | **Evaluated population:**  52 adolescents with ADHD  Age range: 12-16 yrs.  (37 males; 15 females)  **Raters:**  Teachers  Parents | **SCT-14 item scale** [2] | **Diagnostic Interview Schedule for Children-IV** (DISC-IV: [85]): ADHD, psychopathology  **Vanderbilt ADHD Diagnostic Teacher Rating Scale** [153]: ADHD  **Wechsler Intelligence Scale for Children—4^th^ Edition** [106]: IQ  **Homework Problems Checklist** (HPC: [160]): Academic functioning  **Children’s Organizational Skills Scale** (COSS: [111]): Organization, planning, and time-management skills  **Impairment Rating Scale** (IRS: [154]): Behavioral functioning  **School Grades End-of-year grade point average (GPA)**: Academic functioning |
| Lee et al., 2013 [1] | **CB** | **NA** | **Evaluated population:**  366 children  Age range: 5-13 yrs.  (164 males; 202 females)  **Raters:**  Teachers  Parents | **10 item SCT Scale** **of Kiddie-Sluggish Cognitive Tempo Diagnostic Interview Module for Children and Adolescents** [19]: “Easily distracted”, ”Avoids mental effort”, “Does not seem to listen”, “Does not follow instructions”, “Difficulty sustaining attention”, “Difficulty organizing tasks/activities”, “Loses things”, “Forgetful”, “Daydreams”, “Sluggish”. | **Child and Adolescent Disruptive Behavior Inventory** (CADBI: **[**161]): Anxiety, depression, ADHD, ODD, academic functioning, social functioning  **Teacher version of the Child and Adolescent Disruptive Behavior Inventory** (CADBI: **[**161]): Behavioral functioning  **Parent version Child and Adolescent Disruptive Behavior Inventory** (CADBI: **[**161]): Behavioral functioning, anxiety, depression |
| Moruzzi et al., 2013 [26] | **CB** | **NA** | **Evaluated population:**  398 twin pairs  Age range: 8-17 yrs.  (374 males; 422 females)  **Raters:**  Mothers | **SCT items of CBCL 6-18** [162]: “Confused or seems to be in a fog”, “Daydreams or gets lost in his/her thoughts”, “Stares blankly” and “Underactive, slow moving, or lacks energy” | **CBCL** [35]: Behavioral functioning |

C= Clinical population, CB= Community-based population, ON= Some or all participants on medication/ stimulants, OFF= participants off medication/medication naïve participants, MW= Medication withhold at least 24 hr. prior to testing, MU= Medication status unknown/not stated by the authors, NA= Not applicable/no clinical study, ^a^ The sample information presented in the table might differ between studies, since the original studies provided different details and variables, ^b^ Caveat: in this column, labels of scales are listed as used by the authors of the respective studies. Similar/same label were used by authors despite differences concerning the items included.
